# Aetiology and prevalence of subclinical mastitis in dairy herds in peri-urban areas of Kigali in Rwanda

**DOI:** 10.1007/s11250-019-01905-2

**Published:** 2019-04-28

**Authors:** Jean Baptiste Ndahetuye, Ylva Persson, Ann-Kristin Nyman, Michael Tukei, Martin Patrick Ongol, Renée Båge

**Affiliations:** 1 Reproduction, Department of Clinical Sciences, Swedish University of Agricultural Sciences (SLU), P.O. Box 7054, SE-750 07 Uppsala, Sweden; 2 College of Agriculture, Animal Sciences and Veterinary Medicine, University of Rwanda, P. O. Box 210, Musanze, Rwanda; 3 National Veterinary Institute, SE-751 89 Uppsala, Sweden; 4 Växa Sverige, P.O. Box 30204, SE-104 25 Stockholm, Sweden

**Keywords:** Beta-lactamase production, Risk factors, California Mastitis Test, Staphylococci

## Abstract

The aim of this cross-sectional study was to evaluate the prevalence of subclinical mastitis (SCM) and associated risk factors in dairy cows in peri-urban areas of Kigali, Rwanda, and identify causative udder pathogens. A sample of 256 cows from 25 herds was screened with the California Mastitis Test (CMT), and udder quarters with CMT score ≥ 3 (scale 1–5) were milk sampled for culture and final bacteriological identification with matrix-assisted laser desorption ionization time-of-flight mass spectrometry (MALDI-TOF MS). All resultant staphylococci species were tested for beta-lactamase production with the clover leaf method. In parallel, herd bulk milk somatic cell count (SCC) of each herd was analysed using a portable device, the DeLaval cell counter. The prevalence of SCM was 43.1% at quarter level and 76.2% at cow level based on CMT test. Multiparous, Holstein cows were 2.50 (C.I = 1.32–4.71) and 10.08 (C.I = 1.54–66.13) times more likely to contract SCM infection than primiparous animals or cows of other breeds, respectively. The median and mean SCC of all herds were 1108 × 10^3^ cells/mL and 1179 × 10^3^ cells/mL, respectively. The most prevalent pathogens were non-aureus staphylococci (NAS; 40.2%) followed by *Staphylococcus aureus* (22%) and less prevalent pathogens (6%). Samples with no growth or contamination constituted 30.4% and 1.4% of the diagnoses, respectively. The most prevalent species within NAS were *S. epidermidis* (38.2%) followed by *S. sciuri* (19.5%), *S. chromogenes* (9.8%), and nine less prevalent NAS species (32.5%). Out of 209 staphylococci isolates, 77% exhibited beta-lactamase production. The study shows that there is high prevalence of SCM and high herd bulk milk SCC in herds in Kigali, indicating udder health problems in dairy cows. Additionally, beta-lactamase production among staphylococci species was common. Improved milking hygiene and application of biosecurity measures, or a complete mastitis control plan, is required to lower the prevalence of SCM and minimize the spread of pathogens among dairy cows.

## Introduction

Bovine mastitis is a common disease of high economic importance in dairy herds worldwide. The disease can be clinical—with visible signs of illness and milk abnormalities—or subclinical—without visible clinical signs and is affected by several environmental, animal, and management factors (Gruet et al. 2001; Ruegg 2017). Subclinical mastitis (SCM) is 15 to 40 times more prevalent than clinical mastitis (Seegers et al. 2003), and infected cows become major source of infection for healthier cows (Ruegg 2017). This happens when there is lack of implementation of preventive measures such as not wearing gloves during milking (Plozza et al. 2011) or not milking mastitis-infected cows last (Abebe et al. 2016). Cows with SCM have a reduced milk yield and produce milk of lesser quality (Bobbo et al. 2017), which affects farm revenues. Although it is difficult to harmonize methods used in evaluating losses due to both forms of mastitis, the total cost per cow per year was estimated to be 338 € in Sweden in 2010 (Nielsen et al. 2010), 240 € in the Netherlands between 2005 and 2009 (Van Soest et al. 2016), and $ 117.35 in the USA in 1979 (Blosser 1979). Economic estimations of losses associated with mastitis in Africa are rare and not well documented (Motaung et al. 2017); thus, there is an overall low motivation for its control.

Mastitis is a problem for the dairy industry in East Africa where the prevalence of SCM was 86.2% in Uganda (Abrahmsén et al. 2014), 75.9% in Tanzania (Karimuribo et al. 2008), and 62% in Ethiopia (Mekonnen et al. 2017). Mastitis is particularly a concern in Rwanda where there has been importation of high milk yield breeds, which are susceptible to mastitis, without parallel establishment of a mastitis control program (TechnoServe 2008). Mastitis is probably one of the factors that hinder the increase in net milk production yield in East Africa region (Motaung et al. 2017).

Several microorganisms are implicated in mastitis infection; some are environmental pathogens, whereas others are contagious pathogens (Ruegg 2017). The relative importance of each mastitis pathogen varies greatly according to the prevailing management practices of specific countries, or specific regions within the same country, and can change over time (Myllys et al. 1998). Successful mastitis control programs rely on accurate knowledge of the prevalent pathogens. Recently, MALDI-TOF MS has provided speed and reliability in routine analysis to identify mastitis pathogens in developed countries (Nonnemann et al. 2019). The relative importance of mastitis pathogen profile in SCM has not been comprehensively estimated in Rwanda.

Mastitis is the foremost disease that leads to the use of antimicrobials on dairy farm (Menéndez González et al. 2010). Imprudent use of antimicrobials can lead to a rise in antimicrobial resistance (AMR) (Kapoor et al. 2017), and there is risk of subsequent spread of resistant genes to other microbial populations (Xinglin et al. 2017). Resistance has been reported among mastitis pathogens where, for example, the frequency of resistance of *Staphylococcus* (*S.*) *aureus* isolates to penicillin was 47% in Italy (Moronic et al. 2006), 52% in Finland (Pitkälä et al. 2004), and 88% in Tanzania (Suleiman et al. 2018). Björk et al. (2014) reported that 80% of the investigated non-aureus staphylococci (NAS) isolates in Uganda were resistant to penicillin through beta-lactamase production. These levels of resistance could lead to failure of bacteriological cure of mastitis infections (Barkema et al. 2006). Therefore, there is a need not only to determine the level of resistance among mastitis pathogens, for monitoring purposes, but also to guide mastitis prevention and treatment strategies and to detect emerging AMR.

Despite mastitis being a problem for the dairy industry in Rwanda, knowledge about mastitis that could allow a customized control program remains limited. The aim of this study was to evaluate the prevalence of SCM and associated risk factors on cow level in dairy herds located in peri-urban areas of Kigali, in Rwanda. Additionally, the study aimed to identify causative udder pathogens and to evaluate the ability to produce beta-lactamase among resultant staphylococci species.

## Materials and methods

### Description of study area

The study was conducted in peri-urban areas of Kigali located in the geographic centre of Rwanda (− 1° 56′ 22.79″ S 30° 03′ 20.40″ E), surrounding the capital city. Herds located in peri-urban Kigali have higher herd size than the national average, and producers are directly linked to milk consumers or milk processors in the city of Kigali (TechnoServe 2008; MINAGRI 2013). Rwanda has a temperate tropical climate characterized by two seasons. The dry season happens in two periods of the year: the first from June to September and the second from December to February. The other season is wet, which also appears in two separate periods of the year: from February to June and then from September to December. Typical daily temperatures in Kigali range between 15 and 28 °C over the year (David 2007).

### Study design

The study was reviewed, approved and performed in accordance with the ethics operational guidelines and the policy of the University of Rwanda, College Research Screening and Ethics Clearance Committee (RSEC-C) of the College of Agriculture Animal Sciences and Veterinary Medicine, University of Rwanda (UR-CAVM). The ethical guidelines of UR-CAVM were designed in accordance with international standards. Sample size was determined according to Dohoo et al. (2009) as follows:

$$ n=\frac{z^2p\left(1-p\right)}{L2} $$

where:

*n*: sample size, 1.96 = the value of *Z* at 95% confidence interval
*P*: expected prevalence
*L*: desired absolute precision

Therefore, the sample size was determined at 95% confidence level, 6% precision, and with an expected prevalence of 52% from a previous study in Rwanda (Iraguha et al. 2015), thus yielding a sample size of 266 dairy lactating cows. The trained research team and local veterinarian of each subregion used a snowball sampling technique, as described by Faugier and Sargeant (1997), to locate the dairy herd to be enlisted in the study to the north, east and west relative to the centre of Kigali city. A herd was recruited if it had a minimum of five lactating cows, and the herd owner agreed to participate in the study. If a herd had more than 20 lactating cows, only 50% of the cows were included in the study. In total, 256 lactating cows from 25 herds kept in Kigali peri-urban areas were successfully examined. Herds were visited once between May and September 2016.

### Screening for mastitis, milk sampling and bulk somatic cell count measurements

Udder quarter milk samples were collected during ongoing milking by selected trained personnel. For each udder half, the first two or three strips of milk were inspected for milk abnormality and discarded, followed by CMT testing. Subclinical mastitis prevalence was evaluated by CMT using the Scandinavian scoring system (grades 1–5), where 1 indicates negative result (no gel formation, no indicative colour change), 2 is traceable (possible infection) and 3 or above indicates a positive result where 5 has the most gel formation and deep blue/violet colour change (Schalm et al. 1971; Saloniemi 1995). A cow was defined as positive for SCM if she had at least one positive quarter (≥ 3), with no signs of illness and/or visible inflammatory signs of the udder and without visible abnormality in milk. Quarters with CMT ≥ 3 were recorded and sampled for bacteriological analyses according to the National Mastitis Council (NMC) (2017). After cleaning the teat ends with 70% alcohol, an aseptic milk sample was collected in a 10-mL sterile tube and samples were placed and transported on ice inside a cooler box to the microbiology laboratory of the University of Rwanda, College of Science and Technology, Nyarugenge Campus for culture and identification of SCM causative agents (NMC 2017). In parallel, bulk milk samples were collected from each herd and transported in the same manner to the laboratory for SCC analysis with the DeLaval Cell Counter (DCC; DeLaval International AB, Tumba, Sweden). Potential animal factors related to mastitis including breed (three categories: Holstein, Holstein local cross–breed, and other breeds which in turn included local breed, cross-breeds between Jersey and local breeds, and cross between Sahiwal and local breeds), parity (primiparous versus multiparous), stage of lactation (three categories: < 3 months in days in milk (DIM); 4 to 7 months in DIM; > 7 months in milk), calf suckling (yes versus no) and milk production (as continuous variable) were recorded at the time of herd visit through an interview with the herdsman and by observation.

### Bacteriological analyses

All milk samples were cultured on blood agar plates (5% bovine blood with 0.5% esculin) and incubated aerobically at 37 °C for 24 to 48 h before final examination. To be classified as a positive bacterial growth, at least one colony-forming unit (CFU) was needed for the following major pathogens: *S. aureus*, *Streptococcus* (*Str.*) *uberis*, *Str. agalactiae*, and *Klebsiella* spp., and at least five CFUs for the other genera. Samples were classified as contaminated if two or more bacterial types were isolated from one milk sample and growth of the mentioned major pathogens was not identified. If growth of a major udder pathogen was found in combination with contaminating species and the CMT was high, the sample was diagnosed as positive for growth of a major pathogen. Positive isolates were initially characterized based on colony morphology; α-, β-, or double hemolysis; and Gram reaction. Gram-positive isolates were further subjected to catalase and coagulase tests. All isolates, except 45 NAS (out of 169 NAS), were preserved in agar tubes and brought to an accredited laboratory, which is the National Veterinary Institute (SVA: accreditation number 1553 ISO/IEC 17025) in Uppsala, Sweden, for final identification of causative organisms at species level using MALDI-TOF MS. At SVA, each bacterial sample was first re-cultured on horse blood agar, and material from single pure colonies was spotted on a MALDI-plate without pre-treatment. The spots were covered with 1 μL matrix solution consisting of α-cyano-4-hydroxycinnamic acid (HCCA). Subsequently, isolates on MALDI-plate were analysed by the MALDI Biotyper system (Bruker Daltonics, Bremen, Germany) to identify the species. Mass spectra were compared against 4613 spectra in the MALDI Biotyper database using the MALDI Biotyper 3.0 Real-time Classification (RTC) software (Bruker Daltonics, Bremen, Germany). The identification and classification of udder pathogens were done according to MALDI-TOF MS spectra score where a score of ≥ 2.0 was considered reliable identification at species level, a score of ≥ 1.7 to < 2.0 was considered reliable identification to genus level and a score of < 1.7 was considered as no identification. All Staphylococcal isolates were examined individually for beta-lactamase production by the “clover leaf” method as described by Bryan and Godfrey (1991). For quality control, the strains *S. aureus* ATCC 29213 and *S. aureus* ATCC 25923 were used. Identified isolates were stored in trypticase soy broth containing 15% glycerol at − 80 °C.

### Data analysis

The data were recorded in a Microsoft Excel spreadsheet before statistical analysis. The prevalence of SCM was calculated as the number of mastitis-positive cows (one or more quarters with SCM) divided by the total number of cows tested. The quarter SCM prevalence was calculated as number of quarters with SCM divided by the total number of quarters investigated.

To evaluate risk factors associated with SCM, unconditional associations between each independent variable (breed, parity, stage of lactation, calf suckling and milk production) and the dependent variable, cow SCM status (0 = negative and 1 = positive) were investigated using univariable logistic regression analysis. Statistical significance in this step was assessed at *P* value < 0.2. Factors that were significant were then investigated using Spearman rank correlation to assess collinearity, and if two variables showed collinearity (*r* ≥ 0.70), the one with the lowest *P* value was then offered to the multivariable logistic regression model. Initially, the multivariable mixed effect logistic regression model, with herd included as random variable, was used. However, herd as random effect was not significant; thus, the ordinary logistic regression model was used. The multivariable logistic regression model was reduced using a manual, stepwise backward variable selection procedure, where the initial model included all independent variables as main effects. Variables with a significant association (*P* ≤ 0.05) with the dependent variable were kept in the model. All plausible two-way interactions between the significant main effects were tested in the final models. Model fit was assessed by Hosmer–Lemeshow goodness-of-fit test. The statistical analyses were performed using SPSS, version 20.0 for Windows (IBM Corp, Released 2011, IBM SPSS Statistics for Windows, Version 20.0. Armonk, NY: IBM Corp.).

## Results

### Descriptive data

The studied cows had an average milk production of 9.5 L (range 2–22 L) per cow per day. One herd used milking machine; the remaining herds were milked by hand. All cows were milked twice a day. None of the visited herds screened cows regularly for SCM with CMT or included dry cow therapy in their management practices. Approximately 107, 138 and 11 cows were kept in zero-grazing, semi zero-grazing and grazing systems, respectively. The mean herd size was 35 (range 6–200), whereas the median was 22 head (range 5–40) of cattle (lactating cows, dry cows, heifers and weaned calves, inclusive) and the median of lactating cows was 10. There were 2 cows of local breed, 209 cows of cross-breeds between Holstein and local breeds, 3 cows of cross-breeds between Jersey and local breeds, 41 cows of Holstein (pure breed) and 1 cow that was a cross between Sahiwal and local breeds, among the sampled cows.

In total, 256 cows in 25 herds (with 4–20 screened cows per herd) were studied, and 1024 udder quarters were examined. However, there were 20 blind quarters, resulting in 1004 quarters being properly screened for SCM. At the time of quarter sampling, one herd had only four lactating cows. Additionally, 12 quarters from another herd were screened with CMT but sampling was not possible due to cows’ behaviour. In total, 418 quarter milk samples were cultured for bacteriological analyses.

### Prevalence of subclinical mastitis and herd bulk milk somatic cell count distributions

Cow level prevalence of SCM was 76.2% (195 of 256), whereas quarter level prevalence was 43.1% (433 out of 1004). Out of 195 cows with SCM, 129 cows had more than one udder quarter affected. Subclinical mastitis was more common in rear quarters (46.1%; 230 out of 499) than in front quarters (40.1%; 203 out of 505). Median of quarter CMT was 2 (range 1–5). The median and mean bulk-milk SCC for all herds visited was 1108 × 10^3^ cells/mL (central range = 760–1531 cells/mL) and 1179 × 10^3^cells/mL (± 534 cells/mL), respectively. Only two herds had bulk-milk SCC below the SCC limit of 400 × 10^3^ cells/mL, a level set according to EC regulation 853 (EC 2004) regarding herd milk intended for use as liquid milk and human consumption. The herd with the lowest bulk-milk SCC had 352 × 10^3^ cells/mL, and the herd with the highest bulk-milk SCC had 2196 × 10^3^ cells/mL.

### Distribution of udder pathogens

Of the 418 cultured quarter milk samples, 127 (30.4%) were bacteriologically negative; the rest (69.6%) of the quarter milk samples were bacteriologically positive. The most common bacteriological finding was NAS (40.2%) followed by *S. aureus* (22%). Less common were *Acinetobacter iwoffi* (1%), *Enterococcus faecium* (0.7%), *Str. uberis* (0.7%), *Str. agalactiae* (0.7%), *Klebsiella* (*K.*) *oxytoca* (0.5%), *K. variicola* (0.5%), *Pseudomonas rhodesiae* (0.2%) and lastly *P. fluorescence* (0.2%). All above-mentioned species had MALDI-ToF M.S. spectra ≥ 2.0. *Staphylococcus* spp. that were identified at genus level were 1.2% (MALDI-ToF M.S. spectra: 1.7 ≤ score < 2), whereas contaminated samples were 1.4%. Only 0.2% of isolates were not identified by MALDI-ToF MS (score < 1.7). Of the 168 NAS, 123 were subtyped, and the most prevalent NAS species was *S. epidermidis* (38.2%), followed by *S. sciuri* (19.5%). Less common species were *S. chromogenes* (9.8%), *S. xylosus* (8.9%), *S. haemolyticus* (7.3%) and *S. capitis* (6.5%). Other less prevalent NAS species included *S. saprophyticus* (3.3%), *S. pasteuri* (2.4%), *S. devriesei* (1.6%), *S. kloosii* (0.8%), *S. lugdunensis* (0.8%) and *S. warneri* (0.8%). Out of 129 cows that had more than one udder quarter with SCM, 44.9% (58 out of 129) were also bacteriologically positive in more than one quarter. Furthermore, 65.5% (38 out 58) had at least the same bacteriological finding in two of the infected quarters.

### Animal factors associated with subclinical mastitis

Data on prevalence of SCM in each category of selected potential risk factors are presented in Table 1. Initially, mixed effects logistic regression model was used, but as the random effect was not significant, the ordinary logistic regression model was used. Individual factors associated with SCM in the univariable logistic regression analysis (*P* ≤ 0.20) were breed, parity, stage of lactation and calf suckling. There was no statistical evidence of any high correlations among these variables. A significant (*P* < 0.05) association was seen between parity and SCM as well as between breed and SCM. Multiparous cows had significantly (*P* < 0.05) higher odds to contract SCM compared to primiparous cow; Holstein cows and Holstein cross with local breeds had higher odds for SCM than cows of other breeds (Table 2). Hosmer–Lemeshow goodness-of-fit test suggested that the model fit the data (*P* = 0.98).

**Table 1:** Descriptive statistics and results from univariable logistic regression analysis of associations between animal-level factors and the prevalence of subclinical mastitis defined as a score of ≥ 3 in the California Mastitis Tests in 256 dairy cows in peri-urban areas of Kigali, Rwanda

Factors	CMT negative	CMT positive	SCM prevalence (%)	*P* value
Breed				0.077
Holstein	5	36	87.8	
Holstein local cross breed	49	160	76.6	
Others	3	3	50.0	
Parity				0.012
Primiparous	24	48	66.7	
Multiparous	33	151	82.1	
Stage of lactation				0.106
Early lactation (1 to 3 months)	21	51	70.8	
Mid (lactation four to seven)	29	103	78.0	
Late lactation (equal and above eight)	7	45	86.5	
Milk production (mean in L per day)	9.1	9.6		0.464
Calf suckling				0.039
No	18	93	83.8	
Yes	39	106	73.1	

Others = Jersey local crossed breed, Sahiwal breed and local breed

*CMT* California Mastitis Test

**Table 2:** Final multivariable logistic regression model describing the association between risk factors and subclinical mastitis defined as a score of ≥ 3 in the California Mastitis Tests in 256 dairy cows in peri-urban areas of Kigali in Rwanda

Factor	B	S.E	O.R	95% C.I.	*P* value
Parity					
Primiparous	Ref				
Multiparous	0.92	0.32	2.50	1.32–4.71	0.005
Breed					0.048
Other	Ref				
Holstein cross with local breed	1.49	0.84	4.44	0.85–23.15	0.077
Holstein	2.31	0.96	10.08	1.54–66.12	0.016

Other = local breed, cross breeds between Jersey and local breeds, cross between Sahiwal and local breeds

*OR* odds ratio, *C.I.* confidence interval

### Beta-lactamase production among staphylococci species

In total, 209 staphylococc*i* isolates were individually tested for beta-lactamase production. Based on the clover leaf method, 77% (161of 209) of tested isolates exhibited phenotypic resistance to penicillin and thus were reported as positive. Beta-lactamase production was most prevalent in *S. xylosus*, *S. aureus* and *S. pasteuri* isolates (Table 3).

**Table 3:** Prevalence in beta-lactamase production (β±) among staphylococci (S.) isolates from cases of subclinical mastitis in dairy cows in peri-urban areas of Kigali in Rwanda

	Total isolates	Positive	β+ (%)	Negative	β− (%)
*S. aureus*	89	78	87.6	11	12.4
*S. epidermis*	46	34	73.9	12	26.1
*S. sciuri*	23	12	52.2	11	47.8
*S. chromogenes*	9	6	66.7	3	33.3
*S. xylosus*	9	9	100	0	0.0
*S. captis*	8	5	62.5	3	37.5
*S. haemolyticus*	8	6	75.0	2	25.0
*S. pasteuri*	8	7	87.5	1	12.5
*S. saprophyticus*	4	2	50.0	2	50.0
*S. devriesei*	2	1	50.0	1	50.0
*S. kloosii*	1	0	0.0	1	100
*S. lugdunensis*	1	0	0.0	1	100
*S. warneri*	1	1	100	0	0.0
Total	209	161	77.0	48	23.0

## Discussion

The findings of this study showed that the prevalence of SCM (76.2%) and bulk-milk SCC was high in herds in the Kigali region. This is higher than the prevalence levels of below 15% that can be achieved through appropriate udder health care (Ruegg and Pantoja 2013) or 52% (Iraguha et al. 2015) and 50.4% (Mpatswenumugabo et al. 2017) previously reported in north-west and east of Rwanda, respectively. The overall high SCM prevalence may be due to the lack of mastitis control practices, such as the five-point mastitis control plan, which has proved to decrease the prevalence of mastitis and lower bulk milk SCC in the last decades in developed countries (Hillerton et al. 1995). The zero-grazing system particularly prevalent in Kigali (MINAGRI 2013) favours increased infection pressure around the cow, especially when a mastitis control plan is not implemented, resulting in a higher chance of infection in cows in Kigali than in other regions where “non-zero” grazing systems are prevalent. Low farmers’ awareness of SCM (FAO 2014) and the lack of quality or payment standards for bulk milk SCC in Rwanda could explain a low motivation to improve management practices known to prevent and control mastitis, which probably explain the high level of SCM prevalence found in this study.

Our study found that NAS were the most prevalent pathogens causing intramammary infections in SCM cases, followed by *S. aureus*. MALDI-TOF MS used in this study is used routinely for identification of mastitis bacteria at SVA, thus ensuring large library in database for control. This study identified, for the first time in Rwanda, the profile of NAS pathogens involved in SCM at species level. Recent studies conducted in Uganda (Abrahmsén et al. 2014), Ethiopia (Mekonnen et al. 2017) and Rwanda (Mpatswenumugabo et al. 2017) also found that both NAS and *S. aureus* were prevalent in intramammary infections. The increasing importance of NAS in intramammary infections is also reflected in their high prevalence in other geographic regions, such as Germany (Tenhagen et al. 2006), Sweden (Persson et al. 2011), Belgium (Piessens et al. 2011) and the USA (Schukken et al. 2009). The spread and reservoirs of each NAS species depend on several factors including housing systems, management factors, herd size and climate (Nyman et al. 2018). For example, *S. epidermidis*, which is an udder-adapted pathogen, has been isolated on human skin and can spread from cow to cow (Thorberg et al. 2006; Sawant et al. 2009; Piessens et al. 2011). These conditions, together with suboptimal hygiene at farm level in Rwanda (TechnoServe 2008), suggest that *S. epidermidis*, found as the most prevalent NAS species in this study, could have originated from milkers’ hands and spread from cow to cow. Similarly, *S. chromogenes* could have originated from either the udder or the milker’s hands, since these sites are known to be reservoirs of the pathogen and, rarely, the environment. On the other hand, *S. haemolyticus* may have originated from the environment since it is principally an environmental mastitis pathogen (Piessens et al. 2011).

The cause of the high prevalence of *S. aureus* reported in this study could be multifactorial. Beside unhygienic hand milking and lack of a mastitis-control plan mentioned earlier, contagious spread of *S. aureus* in the studied herds could also have been facilitated by not milking mastitis-infected cows last. This practice was found to be associated with mastitis in Ethiopia (Abebe et al. 2016), where conditions are likely similar to Rwanda. Since culling of *S. aureus*–infected cows is not practiced in Rwanda, this implies that the pathogen found in SCM cases in studied herds might have originated from chronic or persistent infections. Major environmental pathogens were less frequent in SCM cases in the studied herds; thus, measures need to focus on combating contagious pathogens. It is worth to note that SCM may be present despite negative bacteriological culture. This may be due to low numbers of bacteria below the limit of detection in the sample, concurrently with a time of sampling when the immune system has successfully eliminated infection before decreasing SCC or with organisms being shed intermittently (Östensson et al. 2013).

The higher risk of SCM associated with increased parity in this study may be attributed to the fact that multiparous cows in the studied herds might have had cumulatively several exposures to mastitis pathogens from suboptimum hygiene during milking or from the environment. Poor integrity of the teat canal due to ageing that leads to easy access of bacterial infection to the mammary gland after milking, decreased immunity or a more pendulous udder prone to injury in older cows than younger cows might all have increased the susceptibility of the older animals to mastitis (Suleiman et al. 2018). It has been reported that the Holstein breed is susceptible to mastitis (Bludau et al. 2014). The higher prevalence of SCM in the Holstein breed than in the Holstein-/local cross-breed or in other breeds reported in this study reflects recent trends in the cattle population in Rwanda, where mastitis susceptible breeds, mainly Holstein, have been increasing since 2006 (TechnoServe 2008) without parallel improvement in udder health care practices.

Beta-lactamase production is the most common resistance mechanism in staphylococci against widely used beta-lactam antibiotics (Livermore and Brown 2001). Beta-lactamase production was common in this study with varying levels of prevalence within staphylococci species from SCM cases. Beta-lactamase prevalence was higher is *S. aureus* and *S. xylosus* than in other isolates. The results are markedly higher than levels of beta-lactamase production in selected mastitis isolates in Sweden (38%), in Finland (32%) and in the Netherlands (37%) (Pitkälä et al. 2004; Sampimon et al. 2009; Persson et al. 2011). The high prevalence of beta-lactamase producing udder pathogens might be due to low application of best practices for infectious disease control, such as culling cows infected with beta-lactamase-producing *S. aureus* strains, mastitis testing before animal trade, proper disinfection during hand milking or other biosecurity measures. Active monitoring for beta-lactamase production among mastitis pathogens, with subsequent control of infected cows, is required to counteract spread of beta-lactamase producing isolates within and among herds in Rwanda.

In conclusion, the high prevalence of SCM and dominance of contagious pathogens indicates that infected cows could be the major source of the infection in the studied herds. Some herds had low bulk-milk SCC, which implies that it is possible to have cows with good udder health in Rwanda. The study also reported that most identified staphylococci species pathogens exhibited beta-lactamase production. Application of biosecurity measures, such as grouping and milking mastitis high-risk cows last, good hygiene and raising awareness of a mastitis control plan among farmers, should be incorporated into herd management practices to effectively improve udder health in Rwanda.
